# Emetine Is Not Ipecac: Considerations for Its Use as Treatment for SARS-CoV2

**DOI:** 10.3390/ph13120428

**Published:** 2020-11-27

**Authors:** Martin D. Bleasel, Gregory M. Peterson

**Affiliations:** 1 School of Pharmacy and Pharmacology, University of Tasmania, Hobart, TAS 7001, Australia; martin.bleasel@gmail.com; 2 School of Health Sciences, Faculty of Health, University of Canberra, Canberra, ACT 2617, Australia

**Keywords:** COVID-19, coronavirus, emetine, ipecac, dehydroemetine, treatment, re-purposing, antiviral, anti-inflammatory, toxicity

## Abstract

Emetine is a potent antiviral that acts on many viruses in the low-nM range, with several studies in animals and humans demonstrating antiviral activity. Historically, emetine was used to treat patients with Spanish influenza, in the last stages of the pandemic in the early 1900s. Some of these patients were “black” with cyanosis. Emetine rapidly reversed the cyanosis and other symptoms of this disease in 12–24 h. However, emetine also has been shown to have anti-inflammatory properties and it appears it is these anti-inflammatory properties that were responsible for the effects seen in patients with Spanish influenza. Emetine, in the past, has also been used in 10s to 100s of millions of people at a dose of ~60 mg daily to treat amoebiasis. Based on viral inhibition data we can calculate a likely SARS-CoV2 antiviral dose of ~1/10th the amoebiasis dose, which should dramatically reduce the risk of any side effects. While there are no anti-inflammatory dose response data available, based on the potential mode of action, the anti-inflammatory actions may also occur at low doses. This paper also examines the toxicity of emetine seen in clinical practice and that seen in the laboratory, and discusses the methods of administration aimed at reducing side effects if higher doses were found to be necessary. While emetine is a “pure drug” as it is extracted from ipecac, some of the differences between emetine and ipecac are also discussed.

## 1. Introduction

We previously discussed the potential use of emetine for the treatment of the SARS-CoV2 coronavirus, at 1/5th to 1/10th the amoebiasis treatment dose [1]. At the time of writing that article it was not definitively known if emetine was active against SARS-CoV2. At least three studies have now confirmed that SARS-CoV2 is sensitive to emetine [2,3,4], with a half-maximal effective concentration (EC_50_) of ~0.5 µM. As discussed in the Supplementary File, this has enabled the calculation of a treatment dose of 6 mg daily, which is 1/10th the normal amoebiasis dose.

Emetine was an important and effective anti-infective drug for the treatment of intestinal and extraintestinal amoebiasis, between the mid-1910s to the 1970s. It was likely administered to 10s–100s of millions of people in the treatment of amoebiasis (see Supplementary File). It was included in the WHO Essential Medicine List until 1983 [5] and the emetine injection is still listed in the United States Pharmacopeia [6]. Despite this, relatively few medical professionals today are familiar with it. A quick online search reveals that emetine is extracted from ipecac. All would have heard of ipecac, and its ability to induce emesis (vomiting). Most would know about the abuse potential of Ipecac Syrup in those with Bulimia Nervosa, as well as Anorexia Nervosa, and that chronic dosing over months can lead to cardiac toxicity and eventually death [7,8].

However, emetine is not ipecac. Ipecac consists of the dried rhizome and roots of *Cephaëlis acuminata,* or of *Cephaëlis ipecacuanha* [9,10]. There were at least 16 different alkaloids isolated from these plants [11]. Emetine, an ether soluble alkaloid, is generally less than 2% by weight of the dried ipecac root [12,13,14]. Early animal studies demonstrated that emetine was inappropriately named. Cephaeline, the other ether soluble alkaloid extracted from ipecac, was found to be approximately twice as emetic as emetine [15]. The United States Pharmacopeia (USP) specifies that the content of cephaeline in “*Ipecac Oral Solution*” (Syrup of Ipecac) can range from an amount equal to, to an amount not more than 2.5 times, the content of emetine [16]. If these were the only alkaloids found in ipecac then the majority of the formulation’s nauseating/emetic properties could be attributed to cephaeline and not emetine. As an illustration, the first patient who was administered emetine by hypodermic injection was a 29-year-old Japanese patient critically ill with amoebic dysentery and who could not tolerate ipecacuanha—the standard treatment at the time. She was administered an emetine dose equivalent to that contained in “*75 grains of ipecacuanha, in sixteen and a half hours, without the slightest unpleasant effect on the patient, who had been unable to retain 1 grain* (of ipecacuanha) *when administered by mouth*” [17].

Emetine was shown to have significant antiviral and anti-inflammatory properties [18], and has the potential to be a safe and effective antiviral agent at low doses. Based on the response of patients with Spanish influenza treated with emetine (described below), it is perhaps the anti-inflammatory properties of emetine that hold the most promise.

## 2. Emetine as an Antiviral

There is no question that emetine has potent antiviral actions, as exhibited by the numerous in vitro studies on multiple viruses demonstrating EC_50_ values in the sub-µM and low-nM ranges, see Table 1. It protected 67% of mice injected with 1000 times the mean lethal dose for 50% (LD_50_) of mouse-adapted Ebola virus [19] and all mice injected 25 times the LD_50_ of enterovirus, EV-A71, with the emetine group showing a million-fold reduction in viral titers in the fore and hind limbs [20]. As expected, all control animals died in both experiments.

Common structural features used to classify viruses, such as enveloped, non-enveloped, DNA or RNA genome viruses, do not appear to affect sensitivity to emetine. Coronavirus, Rift Valley fever, Dengue, Zika and Ebola viruses are all enveloped viruses [21,22,23,24,25] that are sensitive to emetine. Similarly, the numerous enteroviruses that are non-enveloped are also sensitive to emetine [26]. Herpesviruses, double-stranded DNA viruses [27,28,29,30], are as sensitive to emetine as the single-stranded RNA viruses such as Enterovirus, Coronavirus, Rift Valley fever, Dengue, Zika and Ebola viruses [21,22,23,24,25,26]. Viruses with positive or negative single-stranded RNA (+,−ssRNA) also appear to have similar sensitivities to emetine. The Ebola, Human metapneumovirus and Rift Valley fever viruses, with −ssRNAs [22,25,31], and the Coronavirus, Dengue, Zika viruses, with +ssRNAs [21,23,24], have similar sensitivities to emetine. It is unknown if single-stranded DNA viruses have altered sensitivity toward emetine.

Table 2 summarizes the results of antiviral studies involving emetine in humans. Multiple authors have documented their success with emetine treatment of shingles. Most used doses in the 1.2–3.6 mg range given on alternate days for 3–7 injections. Common themes through the papers were: (1) emetine treatment quickly and permanently relieves pain (pain relief often starts in several hours but can take 24–36 h before it goes away completely); (2) emetine accelerates healing of all skin conditions from the shingles (especially if given early; healing times with emetine were halved in all age groups); and (3) there is a distinct lack of postherpetic neuralgia with emetine therapy [18].

Hanisch 1963 [41] treated 26 patients with epidemic kerato-conjunctivitis (believed to be caused by an adenovirus [42]) with 20–30 mg of emetine subcutaneously every other day for five injections. In 24 patients with strongly impaired vision the visual acuity was considerably improved after five emetine injections (one injection every other day), up to months after the initial infection.

From 1938 to 1967 over 600 cases of viral hepatitis in children aged between 1 and 12 years old were treated with emetine at a dose of 1 mg/kg/day. In 100% of cases clinical cure was obtained between 7 and 14 days. No cases had prolonged illness or relapsed. None of the patients experienced undesirable side effects, but patients had “*immediate favorable effects on appetite and general good feeling*” [43]. 

In the early 1960s, Dr Antonio Fusillo an Italian physician, noticed rapid regression of zoster vesicles in patients being treated with emetine for amoebic dysentery. Through several animal experiments over time he determined that only very low doses of emetine, compared to that used for amoebic dysentery, where required to be an effective antiviral. [44,45]. Over the course of his working life Fusillo treated thousands of patients [46] with various viral conditions, such as herpes zoster, chickenpox, epidemic mumps, hepatitis, influenza and lymphocytic meningitis, viral respiratory infections, measles, orchitis epididymitis, and recurrent herpes simplex diseases. The treatment dose he used was predominantly 3 mg every 12 h for the first three doses and then 3 mg per day for up to 10 days depending on the response to treatment [44].

During the two Spanish flu epidemics in New Orleans, in October 1918 and January 1919, Points treated 433 cases of influenza and 73 cases of flu pneumonia [47]. One feature of the epidemic noted by Points was “*extreme cyanosis, amounting to almost blackness*”. Points’ description of his first patient where he noticed the “antiviral” activity of emetine was:

“*Along about January 20 (1919), I treated a case of flu pneumonia complicated by a ruptured appendix. The patient developed acute nephritis, ran 20 per cent of albumen and passed large quantities of blood from the kidneys. Having had some previous experience with intravenous injections of emetine in renal hemorrhages, I injected 1/2 grain* (~30 mg) *emetine hydrochloride into his veins. I repeated this dose in twelve hours and the effect was marvelous, the patient’s temperature dropped from 104° to 101°, the hemorrhage ceased, his cough became better, and his pulse slowed down. The following morning, patient’s temperature was normal and his general condition much improved. Two injections were given that day with the results that patient’s cough subsided, the congestion of the kidneys passed off, the albumen disappeared, his pulse returned to normal and he seemed cured of his toxemia*”

[47]

Points went on to describe the treatment of a group of patients:

“*In the Emetine group I treated 65 cases 9 of which had pneumonia and 2 of which died. All of these nine cases of pneumonia were given from one to two intravenous injections of emetine per day. Purposely no quinin and aspirin were given and no antipyretic used. The result was the immediate lowering of the temperature from “...... the temperature from 104° to 103°* (ranging between 103°–104°) *to normal in from 12 to 24 hours, according as one or two doses of emetine per day were given. The temperature did not rise again and the disease was under control from that time on …… Several of these cases had edema of the lungs and were cyanosed until they were black …… but the results after the intravenous injections of emetine were astonishing. The temperature became normal, the cough subsided, the cyanosis disappeared, and the fight was won ……* (he describes other treatment used) *…… But the remarkable result was the rapid control of the temperature and the toxemia, and the rapid clearing up of the whole picture after the use of emetine*”

[47]

However, there are hints that the response seen with emetine for the Spanish influenza virus, was not due to an antiviral effect, but possibly through acting on the inflammation caused by the virus and not the virus itself:

“*I was so encouraged by the good effects of emetine, when given early enough, that I resolved to use it in all the flu cases, the drug once a day to seventeen simple, uncomplicated cases with these results: The emetine had no effect whatsoever on the non-complicated cases of influenza, not only not reducing the fever, but not controlling any of the symptoms. The disease in these cases ran a course of 3 to 5 days. But none of these cases developed any complications whatsoever from the flu, whereas some of the simple cases, that at first refused the intravenous injections of emetine, later on developed pneumonia and were saved by the drug. One of these simple cases, that refused the injections developed a good attack of cholecystitis, which was instantly relieved by the emetine treatment*”

[47]

If this effect was truly an anti-inflammatory effect, as apposed an ant-viral effect, it is most likely less prone to the development of resistance. Larger studies would have needed to be undertaken to determine if the administration of emetine to uncomplicated cases reduced the likelihood of severe disease. Other noteworthy observations raised by Points:

“*My mortality with the emetine series of cases was virtually nil, for the two cases that did die were beyond hope when the drug was given. …… One had already been sick a week, and her family persistently refused to let me use this treatment until the night before her death; and the other had been sick five days before I got hold of her, and was already well advanced in pneumonia when given the first dose. Even then the beneficial effects of the drug could be seen, the patient’s temperature dropped, and they seemed better for a while, but the delirium and coma steadily grew worse and they passed off.*

*Each case treated with emetine received injections, given from twelve to twenty-four hours apart, according as my time would permit my seeing the patient once or twice a day.*

*Unfortunately I did not use the emetine treatment sufficiently early in the epidemic to determine its true value. The disease was on the wane when I began to use it, so whether or not it would produce the same marvelous results in all cases as it did in these is a question.*

*This problem now presents itself to my mind: Is emetine a cure for the complicated cases of Influenza?*”

[47]

## 3. Antiviral Mode of Action of Emetine 

The mode of action (MOA) of emetine, as an antiviral agent, has not been fully elucidated. However, the MOA may depend on the virus in question. For the coronaviruses, emetine interferes with the viruses’ ability to infect a cell. Shen et al., 2019 performed viral infectivity studies and determined that emetine was an entry inhibitor that blocked MERS-CoV infection almost completely [34]. compared with that of the control, at 5 µM and with an EC_50_ value of 0.16 µM. 

On the other hand, Yang et al., 2018, in order to understand the stage of the Zika virus life cycle affected by emetine, varied compound treatment before, during, and after Zika virus inoculation [19]. For all three stages, emetine was found to have an EC_50_ in the low-nM range (~0.04 µM), partly indicating that the antiviral effect is most likely post-entry and at the step of viral replication [19]. In further support of this argument Yang et al., 2018 also found that emetine directly inhibited the Zika virus NS5 RNA polymerase activity, with an IC_50_ of 0.121 µM [19]. 

As a potential other MOA, Mukhopadhyay et al., 2016, for the human cytomegalovirus (HCMV), found that inhibition by emetine depended on 40S ribosomal processing S14 (RPS14) binding to MDM2. Emetine lead to a disruption of the HCMV-induced MDM2-p53 interaction. HCMV replicated similarly in RPS14 knockdown or control cells, but emetine did not inhibit virus replication in the former cell line. The interaction of MDM2-p53 was maintained in infected RPS14 knockdown cells despite emetine treatment confirming a new mode of action [37].

Tang et al., 2020 demonstrated, by methods described previously [57] that emetine blocks viral IRES-driven translation by the enterovirus EV-A71 [20]. Viral internal ribosome entry sites (IRESs) are unique RNA elements, which use stable and dynamic RNA structures that recruit ribosomes and drive protein synthesis [58]. An increasing number of viruses were shown to initiate protein synthesis by a cap-independent mechanism involving IRESs [20,59]. It is important to note that IRES translation is mediated through the 40S ribosomal subunit [57,58,59]. 

Both Tang et al., 2020 [20] and Mukhopadhyay et al., 2016 [37] demonstrated, by different methods, the key role of emetine’s interaction with the 40S ribosomal subunit in viral inhibition. As the 40S ribosomal subunit is a human protein, there is a potential significant benefit with regards to the development of resistance. Under normal circumstances if an antiviral drug interacts with a viral protein, mutations and the development of resistance can happen quickly in RNA viruses. This is for many reasons, including low-quality replication mechanisms, proofreading, and access to post-replicative repair [20,60]. However, none of these factors affect the human 40S ribosomal subunit as its production is dependent on relatively higher quality replication mechanisms, proofreading, and access to post-replicative repair. Human proteins mutate more slowly than bacterial or viral proteins. IRESs form intricate secondary and tertiary structures with pseudoknots [61,62] that make direct contact with the 40S subunit [61]. Altering RNA sequences can alter the tertiary structure, which offer the possibility of emetine resistance. It remains to be seen if these altered structures can survive in the long term. Tang et al., 2020 [20] noted they could initially induce an emetine-resistant EV-A71 phenotype within nine passages; however, emetine-resistant viruses were not found during long-term treatment in the presence of emetine in cell culture, and mutant viruses did not emerge under the pressure of emetine [20]. Unfortunately, this may not apply to the coronavirus as emetine appears to affect the virus’ ability to infect a cell [34]. This mechanism may be dependent on an interaction with a viral protein that has an increased likelihood of resistance development.

## 4. Actions of Emetine as an Anti-Inflammatory

In the treatment of herpes zoster infections emetine has demonstrated very consistent, rapid, persistent and effective pain relief and wound healing, as demonstrated by numerous authors, at very low doses—in the 2−7 centigrain (~1.2−3.6 mg) dosage range with many patients only requiring 1 or 2 doses and most given under five doses on alternate days [18,49,53,54,56].

Before the antiviral properties of emetine were known, Jorda and Rothschild 1958 [18] set out to determine the mechanism of action of emetine that made it effective in the treatment of herpes zoster infections. First, they tested the hypothesis that emetine was an analgesic by using the “hot plate test”. Forty mice were placed on a glass base heated to 60 °C and the reactivity (attempt to jump up) was assessed with and without emetine. Despite the high dose used, emetine had no effect. Emetine had no analgesic properties [18]. Jorda and Rothschild, in 1958m performed a series of experiments injecting inflammatory substances into the plantar aponeurosis of the rear paw of Wistar rats and monitoring how well emetine reduced inflammation relative to control rats. The three substances were: diluted egg white, hyaluronidase, and formalin. Two hours after the egg white injection emetine (5 mg/kg) had reduced the swelling (*p* ≤ 0.05) relative to control. Emetine also significantly reduced (~34%; *p* < 0.01) the swelling induced by hyaluronidase relative to control. The formalin inflammation experiment was monitored for changes in induced inflammation at multiple time points during the first seven hours and the following 10 days. From this last experiment the anti-inflammatory effects increased and peaked in the seventh hour, closely matching the latency seen in patients treated for shingles [18]. Furthermore, monitoring over 10 days showed significantly less skin ulcerations and swelling in the emetine group than those in the control group [18]. Another study found that emetine (0.05 mg/kg/day; 4 weeks) significantly reduced the secretion of cytokines/chemokines and growth factors (e.g., IL-1β, IL-6, and TNF-α) in Sugen/hypoxia-induced pulmonary hypertensive rats [63].

Several studies looked at the action of emetine on interferon/s and various aspects of the immune system [64,65,66,67,68,69,70,71]. It is beyond the scope of this article to extrapolate data from one or more isolated, “*interferon*” or the like, in vitro, or in vivo studies to explain a clinical observation. Suffice to say that it is well known that emetine inhibits protein synthesis and this action is understood to be responsible for the amoebicidal action of emetine [72,73,74]. It is also known, as described above, that emetine interacts with the 40S subunit of the human ribosome to inhibit viral protein synthesis. It is highly possible that the action of emetine on protein synthesis would affect the production of multiple interferon proteins and other immune-altering proteins. It would not be unreasonable to assume that it is this action, at least in part, that is responsible for the anti-inflammatory activity of emetine.

To further support this argument it was shown that the human mTOR (central regulator of immune responses) [75] transcript can be translated in a cap-independent manner that forms an RNA scaffold capable of binding directly to the 40S ribosomal subunit [76].

## 5. Toxicity of Emetine

The toxicity emetine will be described in terms of the toxicity likely to be seen in clinical practice and the toxicity seen in the laboratory.

### 5.1. Toxicity of Emetine in Clinical Practice

The toxicity of emetine, like most drugs, is dose-related. With a long biological half-life it can accumulate, and that accumulation does increase the risk of toxicity. This toxicity of emetine will be explored in terms of low and high doses of emetine. Low doses will be defined as doses less than 6 mg (with many studies only using ~3 mg) of emetine daily. The majority of studies employ doses of less than 6 mg per day. High doses will be defined as between ~20 mg and 60 mg. When emetine was used widely, the increased risk of emetine toxicity in the elderly/frail was very much recognized and usually warranted a reduction of the amoebiasis dose by at least half (~30 mg or less). In some situations, emetine would have been contraindicated but at times there was little choice, especially with regards to treating liver abscesses.

The information below is not intended to be a replacement for appropriate prescribing information or other drug monographs.

#### 5.1.1. Cardiac Toxicity

*Low dose:* No study referenced in this paper using low dose emetine highlighted a cardiac concern.

*High dose:* Cardiac toxicity has traditionally been the largest concern of emetine therapy. Yang et al. 1980 reviewed the cardiovascular side effects of therapeutic doses of emetine, concluding that cardiac toxicity frequently included ECG changes and hypotension (discussed below) and occasionally tachycardia and precordial pain. These changes occur during treatment or after completion of treatment and often last a period of time. The patient usually recovers without any change in cardiovascular function [77].

ECG changes, particularly flattening or inversion of the T-wave and prolongation of the Q-T interval, occur in many (25−50%) patients [78,79]. However, cardiac disease and renal disease (likely due to the risk of drug accumulation) are generally considered contraindications.

#### 5.1.2. Hypotension

*Low dose*: the only study that mentioned blood pressure at all was Griveaud and Achard 1959 who described the hypotensive effect as minimal and of a short duration (“not durable”) but believed it was important to monitor [49].

*High dose:* It is said that the hypotension from emetine is rarely marked [78]. However, the risks associated with patients who are frail, elderly, and potentially the risk of co-existing hypotension from viral septicemia would be higher. Harinasuta 1951, described a marked fall in blood pressure (no values given) and severe prostration with rapid pulse in 5 out of 45 patients with the majority treated with 30 or 60 mg of emetine daily for amoebic liver abscesses. After intravenous injection blood pressure starts to drop 15−20 min after the injection [80]. Klatskin 1948 reported a fall in systolic pressure of 15 to 20 and the diastolic 5 to 10 mm of mercury in ~30% of patients [81]. In a previous article by Klatskin, in treating over 500 servicemen, he commented “*Occasional patients exhibited a fall in blood pressure, rarely more than 15 or 20 mm. of mercury, which usually occurred between the sixth and ninth doses*” [82]. Unlike low-dose emetine the high-dose effect on blood pressure may be for a longer duration, with Harinasuta pausing treatment for 5 days in one patient and 13 days in another [80].

#### 5.1.3. Nausea

*Low dose:* Very few of the studies using low-dose emetine reported any nausea. Most noted that there was no nausea or did not comment on it at all. Griveaud, reporting on 5 years’ experience with emetine, noted that a few of the patients had some nausea [49]. One study with 31 patients reported that one patient vomited, with all other subjects without the slightest discomfort [54].

*High dose:* The incidence of nausea in patients taking ameobiasis doses of emetine are highly variable. Heilig [83] commented that in his study of 45 patients that: “*not a single case suffered from nausea, vomiting or toxic diarrhea, from neuritis or muscular weakness*” and suggested that “*it is possible that these favorable results are partly due to the absence of toxic contaminations (cephaeline) in the brand of emetine that is used in our hospital*”. On the other hand, Klatskin 1948 [81], with 100 subjects, had one of the highest incidences of nausea: “*Nausea occurred in almost a third of the subjects and was only occasionally accompanied by vomiting. In many it appeared within two hours of an injection and subsided rapidly, but in others it was more persistent*”. Interestingly, this study used several commercial preparations of emetine hydrochloride [81].

Nausea from the ipecac alkaloids is relatively easy to treat. The emetic effects of ipecac syrup (30 mL) can be completely eliminated, and nausea significantly reduced, by the use of 5HT_3_ antagonists, such as ondansetron [84]. Emetine has a high affinity for the 5HT_4_ receptor with little activity on 5HT_3_ [85]. However, it should at least be considered that they could interact until in vitro studies can confirm otherwise.

#### 5.1.4. Pain on Injection

Pain on injection is included because it is a common feature of both subcutaneous and intramuscular injection and for low and high doses of emetine. In relation to intramuscular injections (high dose); pain can be almost immediately after injection and disappear in a few hours, but in the others the pain may not appear until a day or two later. There can be local tenderness, poorly localized, constant aching of the injected muscle, or more commonly, of all the surrounding muscles. The aching and tenderness usually lasts for several days to a week after treatment is stopped. Emetine is rarely discontinued because of pain [82].

### 5.2. Method of Administration and Toxicity

Traditionally, the preferred routes of emetine administration are by deep subcutaneous or intramuscular injection. The intravenous use of emetine was considered contraindicated because it was thought to be too dangerous and offered no therapeutic advantage [78]. However, there are advantages of intravenous and subcutaneous infusions in the intensive care unit (ICU) setting. Infusions given over many hours may reduce the incidence of side effects. If side effects occur, including adverse changes in electrocardiography (ECG) or blood pressure, the rate of administration can be reduced or stopped. It was observed that emetine solutions of less than 0.2%, produce little local effects [86]. A dilution of 30 mg emetine in 20 mL (0.15%; 1/20th of the normal intramuscular concentration) may be able to be administered subcutaneously over many hours. No efficacy or safety data of slow emetine infusions were found. Caution is warranted.

Parmer and Cottrill 1949 observed that soon after injection of emetine in rabbits, the heart levels were relatively high, but after 6 h had dropped off to the low levels seen over the next 6 days [87]. To reduce peak concentrations of emetine in the heart, if high doses need to be given, infusion times of greater than 6 h for 30 mg should be considered, especially in the old and frail.

For low-dose emetine a 6 mg dose could be given, subcutaneously or intramuscularly, in divided doses to minimize risk.

### 5.3. In Vitro Toxicity

Table 1 lists in vitro antiviral studies of emetine with corresponding EC_50_ and, where determined, half-maximal cytotoxic concentration (CC_50_) values and the selectivity index (SI; SI = CC_50_/EC_50_) calculated. The CC_50_ values show a large degree of variation depending on the type of CC_50_ test, cell line and the duration of drug exposure.

There is a large disparity between the toxicity seen in the laboratory to what is seen in practice. For example, looking at the number of studies in Table 1 that have CC_50_ values of 8 µM and less, and comparing those values to the conservative tissue concentrations achieved in Supplementary File, Table S1, there are four major organs that achieve tissue concentrations of emetine over three times the CC_50_.

There are several reasons that could account for these discrepancies. Several CC_50_ tests use a method that indirectly measures metabolic activity (i.e., MTT) or determines cell proliferation (cell counting). It is assumed that a decrease in metabolic activity or a proliferation is an indicator of cell toxicity or death. This is not always the case. Emetine is a protein synthesis inhibitor known to inhibit the 40S subunit of a ribosome [88,89]. This type of drug would reduce metabolic activity, and cellular proliferation but does not necessarily result in cell death. Bacteriostatic antibiotics behave in a similar fashion and reduce bacterial metabolic activity and proliferation without necessarily killing the bacteria. Examples would include the antibiotics doxycycline, chloramphenicol, and erythromycin which also inhibit protein synthesis by interfering with the 30S/50S subunits of the ribosome [90].

Another limitation of CC_50_ tests, in relation to emetine, is the cell lines used to determine toxicity. Emetine is well known to exhibit anti-cancer properties [91,92]. The cell lines used in the CC_50_ are immortal cell lines, arguably sharing several characteristics of cancer. Their sensitivity to emetine could depend on how the cells were immortalized (i.e., isolation from cancer cell, type of viral immortalization). Toxicity seen in these cell lines may not be reflective of what happens to non-cancerous cell lines in vivo. This action is also seen in non-cancer cell lines. Emetine was shown to inhibit hyper-proliferating pulmonary artery smooth muscle cells (PASMCs) from rats with pulmonary hypertension, normalizing proliferation/apoptosis. However, emetine had minimal effect on non-hyper-proliferating PASMCs. This action was hypothesized to be due in part to the normalization of hypoxia-inducible factors under hypoxic conditions [63].

Overall, the value of in vitro CC_50_ and SI data, in the case of emetine as an antiviral, is of little value. Emetine has been used extensively in millions of humans over many years for the treatment of amoebiasis and the more relevant in vivo toxicity is well described and understood. Furthermore, as the antiviral and potentially anti-inflammatory doses being suggested are substantially lower than that used for the treatment of amoebiasis any toxicity will be significantly lower.

## 6. Discussion

This paper, following on from our previous work [1], further explores the use of emetine as an antiviral agent and addresses some preconceived notions that people would have when they first learn that emetine is obtained from ipecac. Furthermore, the paper estimates the antiviral dose required to be effective against SARS-CoV2 and gives a means to calculate the likely effective dose of emetine against other viruses, based on viral inhibitory EC_50_ data (Supplementary File).

The likely antiviral dose for emetine is approximately 6 mg daily (given as a single or divided doses) for SARS-CoV2. At this low dose, the side effects associated with the normal treatment dose of amoebiasis are unlikely to be a clinical problem. Traditional contraindications of kidney or heart disease are also much less likely to be an issue. Based on EC_50_ data, the dose required for the treatment of non-coronaviruses viruses is likely to be substantially lower than 6 mg, reducing the risk of toxicity even further.

The rapid reversal of cyanosis seen with Spanish influenza appeared to be an effect independent of emetine’s antiviral activity. If anything, the Spanish influenza virus was arguably resistant to emetine, with no decrease in temperature or a reduction of any symptoms or shortening of the length of the disease in mild cases. Added to that a patient’s cholecystitis pain being relieved by emetine further suggests an anti-inflammatory response, given that emetine has no analgesic properties [18]. There is growing recognition that the hyperinflammatory response induced by SARS-CoV-2 is a major cause of disease severity and death in the infected patient [93,94,95,96]. If emetine could reduce the inflammatory response in the SARS-CoV2 patient in the same way it appeared to in patients with Spanish influenza, then the anti-inflammatory effect of emetine may be more important than the antiviral effect. This then raises several questions that should be addressed.

At what dose are you likely to see an anti-inflammatory effect? In this article, we hypothesized that the anti-inflammatory MOA is the same as the antiviral and anti-amoebic MOA—through the inhibition of protein synthesis—in particular, binding to the human 40S subunit of the ribosome. If this is correct then the anti-inflammatory doses may be very similar to the antiviral doses (low). However, the effect seen by Points [47] for the treatment of Spanish influenza was seen at 30–60 mg per day.

Is a loading dose necessary? Loading doses were never used for the treatment of amoebiasis. It would not have been possible to safely administer such large doses. For antiviral doses a single large dose could be a loading dose or, because of its long biological half-life, a sustained-release dose. However, it is unknown if RNA needs to be detached from the 40S subunit in order for emetine to attach. If this is the case multiple doses may have a better outcome.

What is the likely duration of therapy? Emetine protein synthesis inhibition is considered irreversible [97,98]. Therefore, not many doses may need to be given. Fusillo, who treated numerous viral diseases at 3 mg every 12 h for the first 24 h and then 3 mg daily, found that 10 days was sufficient for any viral disease [45], but many authors comment on a noticeable improvement between a couple of hours to a few days.

What alternatives are there to emetine? A potential alternative to emetine is the very structurally similar, and completely synthetic dehydroemetine. An injection form of dehydroemetine is still available in India from Tablets India Limited, under the brand name Tilemetin. It is currently unknown if this agent has similar antiviral and anti-inflammatory properties, and it should be tested if emetine is found to be effective. Dehydroemetine is believed to be safer agent than emetine in terms of cardiac toxicity [99]. Glaxo, in the 1960s, developed an oral dehydroemetine resinate preparation that was well tolerated [100,101].

Can emetine be used in combinations with other drugs to improve antiviral efficacy or reduce side effects? The concept of antiviral synergism is well known and understood. With regards to emetine, Choy et al., 2020 demonstrated that emetine and remdesivir, when used in combination, displayed significant antiviral synergism [2]. This implies that these drugs could be used at lower doses to potentially reduce overall side effects without compromising the antiviral efficacy. However, if the dominant therapeutic effect of emetine therapy is an anti-inflammatory effect it could be potentially unwise to reduce the dose of emetine, especially if the anti-inflammatory action is already at a low dose.

If emetine proves to be successful for the treatment of SARS-CoV2, either as an antiviral or an anti-inflammatory agent, then this would place a huge stress on wild populations of *Cephaëlis acuminata* or *Cephaëlis ipecacuanha* from which emetine can be obtained. While these plants are commercially grown, similar wild species/varieties are endangered [12,13]. There simply would not be sufficient plant material to treat all that that would require it. Ultimately, and quickly, emetine would need to be produced commercially and synthetically. Fortunately, this process has been established since the 1960s [102,103,104].

## 7. Conclusions

Emetine, shows promising antiviral and anti-inflammatory properties. The use of low-dose, or even, high-dose emetine should be considered in the treatment of COVID-19. Other viruses are far more sensitive to the effects of emetine than SARS-CoV2, for which emetine may prove useful.

## Figures and Tables

**Table 1:** EC_50_ values of emetine against various viruses. The majority of the viruses have an EC_50_ value in the low-nM range. For comparison purposes the EC_50_ value of Entamoeba Histolytica is included. Most viruses have an EC_50_ value 20–300 times less than Entamoeba Histolytica, indicating that much lower doses would be required for antiviral treatment than the doses used for the treatment of amoebiasis. The half-maximal cytotoxic concentration (CC_50_) and the selectivity index (SI) data is included to demonstrate that the in vitro toxicity, as represented by the CC_50_, varies dramatically and that the concentrations are often substantially lower than the likely concentration achieved in practice see Supplementary File, Table S1. The in vitro CC_50_ appears to have little resemblance to what is seen in practice.

Virus	EC_50_ µM	Cell Line	CC_50_ Assay	CC_50_ µM	SI (b)	Reference	Notes
***Entamoeba Histolytica***							
	11.8						Not a virus, included for comparison and is based on the average EC_50_ data (*n* = 7) from Burchard and Mirelman 1988 [32]
***Coronaviruses***							
SARS-CoV2	0.50	Vero E6	Cell titer-Glo luminescent (Promega)	56.46	112.92	Choy et al., 2020 [2]	
SARS-CoV2	0.52	Caco-2	ATP assay	>20 (see Notes)	>38 (see Notes)	Ellinger et al., 2020 [4]	Tabulated data gave the CC_50_ as 1.13 µM, graphed data did not approach CC_50_ at 20 µM
SARS-CoV2	0.47	Caco-2	Rotitest Vital (WST-8; Roth)	>30	>63	Bojkova et al., 2020 [3]	
HCoV-OC43	0.21	HCT-8	MTS	>50	>238	Yang et al., 2020 [33]	
HCoV-OC43	0.3	BHK-21	MTT assay	2.69	8.97	Shen et al., 2019 [34]	
MERS-CoV	0.34	Vero E6	MTT assay	3.08	9.06	Shen et al., 2019 [34]	
MERS-CoV	0.08	Vero	Method not disclosed	>25	>312.5	Ko et al., 2020 [35]	
MERS-CoV	0.014	Vero E6	Not determined			Dyall et al., 2014 [36]	
SARS-CoV	0.051	Vero E6	Not determined			Dyall et al., 2014 [36]	
HCoV-NL63	1.43	LLC-MK2	MTT assay	3.63	2.54	Shen et al., 2019 [34]	
MHV-A59	0.12	DBT	MTT assay	3.51	29.25	Shen et al., 2019 [34]	
***Herpesviruses***							
Human Cyto-megalovirus	0.04	human foreskin fibroblasts	MTT Assay	8	200	Mukhopadhyay et al., 2016 [37]	
herpesvirus-1	0.056	human foreskin fibroblasts	MTT Assay	8	143	Mukhopadhyay et al., 2016 [37]	
herpesvirus-2	0.033	human foreskin fibroblasts	MTT Assay	8	242	Mukhopadhyay et al., 2016 [37]	
herpesvirus-2	0.03	retinal pigment epithelial cells	Cell Tox Green reagent	1.12	37	Andersen et al., 2019 [38]	
***Enteroviruses***							
Echovirus 1	0.14	retinal pigment epithelial cells	Cell Tox Green reagent	>30	>214	Andersen et al., 2019 [38]	
EV-A71 (hand, foot, & mouth disease in children)	0.049	RD Cells	Cell Counting Kit 8	10	204	Tang et al., 2020 [20]	
EV-D68 (can lead to severe respiratory illness)	0.0187	RD Cells	Cell Counting Kit 8	10	535	Tang et al., 2020 [20]	
Echov-6	0.045	RD Cells	Cell Counting Kit 8	10	222	Tang et al., 2020 [20]	
CV-A16 (hand, foot, and mouth disease in children)	0.083	RD Cells	Cell Counting Kit 8	10	120	Tang et al., 2020 [20]	
CV-B1	0.05	RD Cells	Cell Counting Kit 8	10	200	Tang et al., 2020 [20]	
***Other Viruses***							
influenza A	0.13	retinal pigment epithelial cells	Cell Tox Green reagent	>30	>230	Andersen et al., 2019 [38]	Tang [20] noted emetine had no effect on rotavirus and influenza. Data was not shown, influenza type not given
Human meta-pneumovirus	0.14	retinal pigment epithelial cells	Cell Tox Green reagent	1	7	Anderson et al., 2019 [38]	
Rift Valley fever virus	0.43	retinal pigment epithelial cells	Cell Tox Green reagent	>30	>69	Andersen et al., 2019 [38]	
Dengue virus Serotype-2	<0.5 (see Notes)	Huh-7	alamarBlue reagent (Invitrogen)	>10 (see Notes)	>>20 (see Notes)	Low et al., 2009 [39]	Infection reduced by more than 2.5 log units in cells treated at 0.5 µM; >90% cell viability at 10 µM, therefore SI must be significantly higher than 20.
Zika	0.053	HEK293	Possibly ATPLite assay used in supplement.	0.18	3.4	Yang et al., 2018 [19]	
Ebola	0.017	Vero E6	Not determined	-	-	Yang et al., 2018 [19]	
HIV	<0.03	PBMC	Propidium iodide staining and flow cytometry	0.1	>3.3	Valadão et al., 2015 [40]	Two different cell lines obtained very different results. As the EC_50_ for Ghost Cells is significantly greater than the EC_50_ for *Entamoeba Histolytica*, this would be considered highly resistant
HIV	30–100	Ghost cells	CellTiter-Blue reagent	>360	>3.6	Valadão et al., 2015 [40]	

**Table 2:** Emetine use as an antiviral agent in humans.

Year/s (*)	Condition	Number of Patients	Dosing (**)	Side Effects	Notes	Language [Reference]
1920	Spanish Influenza	10 pneumonia (severe) 17 mild cases	~30 mg IV every 12−24 h; 6 doses	None described	Within 24 h for severe cases: Fall in temperature, reversal of “black” cyanosis, reversal of tachycardia, reduction in coughing patients recovers (see notes within main document). No effect in mild cases at all	English [47]
1937	Shingles	3	Escalating doses on same day ~1.2, 1.8, 2.4 and 3 mg	None described	Clear reduction in pain begins a few hours after first injection, after 2−5 days blisters are parched and the pain disappears completely	French [48]
1952	Shingles	47 [18]	2.4 mg SC alternate days for 2−3 days. Up to 3.6 mg for severe cases [49]	Unknown	This dosage regimen was reported elsewhere [49]	French [50] (^)
1953	Shingles	>30	Unknown presumed low doses given	Unknown	This was a thesis; 30 cases were submitted from another author [49]. As it was a French thesis it was considered impractical to obtain and translate in a reasonable time frame	French [51] (^)
1954	Shingles	>40	Unknown presumed low doses given	Unknown	Was quoted as saying “*cured quickly without any failure*” more than 40 patients [49]	French [52] (^)
1954	Shingles	13	2.4 mg on 1st, 3rd and 5th day	“*No ocular complications were noted*”	Confirming the results of [49] “*the cessation of pain after the first injection of emetine was truly spectacular and very rapid drying of the vesicles since healing takes 8 days was obtained*” [53]	French [53]
1957	Shingles	31	1.2−3.6 mg SC daily, five injections at most	One patient vomited. No other patients had the “slightest discomfort”	One case out of the 31 was considered a failure. While it healed, they believed for this case it followed the natural evolution of the disease	French [54]
1958	Shingles	40	2.4−3.6 mg on 1st, 3rd and 5th day	No side effects mentioned	Paper compered results with his past results and with the literature: (1) quickly and permanently relieved pain (2) accelerated healing of all skin lesions (3) no postherpetic neuralgia in any patient	German [18]
1959	Shingles	160 “areas” treated, patient numbers not given.	2.4−3.6 mg every other day max. 7 injections	Few patients had nausea	Disappearance of cutaneous hyperesthesia (a sign of success). Rapid drying of lesions 3 to 7 days. Rapid regression of lymph node sensitivity. Sleep often possible from the first night	French [49]
1961	Viral aphthous stomatitis	56 children (~90% <6 years old)	1 mg/kg SC up to 10 doses (10 was never reached)	None mentioned	With the application of the first dose the symptomatology quickly subsided—between 3 and 7 days to clinical cure	Spanish [55]
1963	Epidemic kerato-conjunctivitis	31	20−30 mg SC every other day, 5 doses.	None mentioned	In 24/26 patients with strongly impaired vision the visual acuity could be considerably improved after five emetine injections (1 injection every other day)	Hungarian [41]
1964	Ophthalmic Shingles	41	1.2−2.4 mg SC every other day for 4−6 injections	None mentioned	Only six cases had no improvement. Blisters dried quickly, pain resolved and no cases of postherpetic neuralgia. This study noted quick resolution, however appeared more slowly than other studies	Polish [56]
1968	Hepatitis	600 children	1 mg/kg SC for 7−14 days	Perfectly tolerated, no undesirable side effects	100% cure rate, had an immediate effect on appetite and well being	Spanish [43]
1960s−80s	Herpes viruses, hepatitis, influenza, mumps	1000s [46]	3 mg every 12 h for the first 24 h then 3 mg per day	Negligible. In relation to amoebiasis doses it was considered homeopathic	Over the course of his working life Fusillo treated thousands of patients with this dosage regimen, he believed it to be a very broad acting and effective antiviral agent at relatively low doses	Italian [44,45,46]

(*) A single date refers to the publication date. (**) SC = Subcutaneous, IV = Intravenous; doses are a representation, often doses changed on the clinical situation. (^) Reference was unable to be obtained, information was obtained from one or more other articles. Bibliography reference details may not be reliable.

## Supplementary Materials

The Supporting File is available online at https://www.mdpi.com/1424-8247/13/12/428/s1.
