# Supplementary material for: Emetine Is Not Ipecac: Considerations for Its Use as Treatment for SARS-CoV2
Source: Pharmaceuticals (Basel). 2020 Nov 27;13(12):428. doi: 10.3390/ph13120428 (PMC7760625; doi:10.3390/ph13120428)
Supplement: Supplementary file 1 [file pharmaceuticals-13-00428-s001.pdf]

Supplementary file

1. The Extent of Emetine Use

Emetine was a drug used widely. Based on the severity and prevalence of amoebiasis, as well as limited export data, the number of patients treated with emetine between 1912 and the 1970s would be in range of the 10s to 100s of millions of people.

In 1997 the World Health Organisation (WHO) estimated that there were approximately 48 million people with symptomatic amoebiasis, such as amoebic dysentery, from *Entamoeba Histolytica* [1]. In the pre-antibiotic era, approximately 1 case of amoebic liver abscess was found for every 10 cases of amoebic dysentery [2]. Amoebic liver-abscess was a relentlessly progressive and almost invariably fatal disease little more than a century ago [3]. Emetine during the first half of the 20th century was one of the only well tolerated treatments for amoebic liver abscesses, and had a survival rate of approximately 90% [4,5]. In the late 1940s chloroquine begun to be used for the treatment of liver abscesses; however, the cure rate appeared lower and the relapse rate was higher than with emetine [5]. Later recommendations suggested the combination of chloroquine and emetine [6,7]. More choices were available for the treatment of amoebic dysentery; however, emetine was still used widely [8].

Brazil, based on emetine export figures, reached an average annual, potentially peak, output of about 600 kilograms in the 3-year period of 1944 through to 1946 [9]. Assuming a treatment course of 60 mg dose for 7 days, this would equate to approximately 5.7 million courses of emetine given during that time period. The total use in the USA up to this time was estimated to not exceed 30 kg annually [9]. Assuming a conservative 10 kg annual consumption in America between 1920 and 1943 this would equate to more than 500,000 treatment courses in the USA alone over this period.

However, these numbers are low in relation to the likely use of emetine in India. Between 1945 and 1960 it was estimated that between 10-40% of the Indian population suffered from chronic amoebiasis [10]. Grossman 1969, found 57% of approximately 30,000 American Peace Corps volunteers had clinical amoebiasis at least once during their two years in India [11]. If it is assumed that the 1921 population in India remained stable at 319 million [12] for 40 years and only 1% of the lower 10% incidence of amoebiasis (0.1% of the population) were treated, this equates to approximately 13 million courses over 40 years. These figures are likely to be a significant underestimation of the true use of emetine in India.

2. Emetine dose estimation for SARS-Cov2

There is a lack of pharmacokinetic data for emetine in humans. To calculate an antiviral dose we have assumed that the concentration of emetine achieved in the liver reaches a conservative minimum inhibitory concentration MIC of 25 μM at an amoebiasis dose of 1 mg/kg up to 60 mg for up to 10 days [13].

A conservative reference MIC value 25 μM was chosen as emetine alone does not result in a cure of amoebic liver abscesses in 10% of patients [5]. A reference MIC of 25 μM is in the lower quartile MIC range reported by Pedrazzoli *et al* 1971 [14], and is approximately half the concentration, 48 μM, where Burchard and Mirelman 1988 [15] found live *Entamoeba Histolytica*. The 25 μM is just over double the EC_{50} of 11.8 μM found by Burchard and Mirelman whereas Bansal demonstrated only an ~85% inhibition at ~4 times the EC_{50}. The EC_{50} value (26.8 μM) by Bansal *et al* for the reference strain HM1:IMSS was not used as the same strain tested by Burchard and Mirelman 1988 [15], using almost identical cell counting and culturing methods, had an EC_{50} of less than half (12.6 μM; 6.5 μg/ml).

Using a conservative MIC of 25 μM, other tissue concentrations can be extrapolated based on radiolabeled emetine studies in animals. **Table 1** shows the likely tissue concentrations that would be achieved in a human receiving a course of emetine parenterally. While the figures in this table are based on that found in a guinea pig they would be unlikely to be practically different to those found in humans. These results were broadly consistent with a different emetine radiolabeled study in mice and the differences seen were consistent with what would be expected from the different route of administration [16,17]. While the results of spectrophotometric studies are also broadly consistent [18,19] more weight was placed on radiolabeled emetine data [20]. It should be noted that as emetine does not accumulate in the CNS, emetine alone is unlikely to be useful in a CNS infection. From animal models emetine does appear to delay / reduce viral entry and load in the brain [21].

Table 1: Radioactivity expressed as emetine concentrations (μg/g fresh tissue homogenate) in various organs of the guinea pig after 8 daily injections of 1.87 mg of ^{14}C-labeleddrug base per kg. Adapted from [20]. Proportion and theoretical emetine concentrations found in various tissues are then extrapolated based on an MIC of 25 μM in the liver.

Tissue	Radioactive Emetine (μg/g)	Proportion in each tissue	Theoretical tissue concentration (μM)
a) Lungs*	70.26	31.99%	58.82
b) Spleen	57.42	26.14%	48.07
c) Liver	29.86	13.59%	25.00
d) Kidney	39.61	18.03%	33.16
e) Heart	4.99	2.27%	4.18
f) Fat	1.14	0.52%	0.95
g) Adrenals	15.54	7.07%	13.01
h) Muscle	0.66	0.30%	0.55
i) Brain	0.18	0.08%	0.15

*a)-e) = mean of 3 animals; f)–j) = one animal; - not done [20].

Likely doses required to treat SarsCoV2

Three separate studies have confirmed the EC_{50} of SARS-CoV2 is approximately 0.5 μM (see **Table 2**), from **Table 2** it can be seen that the concentration of emetine to have the maximum viral inhibition (EC_{90-100}) is

between 2-5 μM. To be conservative, calculations will be based on 5 μM. On this basis, comparing the MIC of *Entamoeba Histolytica* with the EC_{90-100} the SARS-CoV2 coronaviruses, we can calculate that approximately one-fifth (5 μM/25 μM) of the normal emetine dose would be all that is required to treat SARS-CoV2. Additionally, from **Table 2** it can be seen that the concentration of emetine in the lungs is over twice the concentration in the liver. Therefore, potentially one-tenth (~6 mg) of the *Entamoeba Histolytica* dose could be used to treat a SARS-CoV2 infection. At one-tenth of the *Entamoeba Histolytica* treatment dose this would show a dramatic reduction in side effects, such as the incidence of ECG changes and nausea.

Table 2: EC_{50} values for emetine against the SARS-CoV2 virus from various studies. The study by Weston et al 2020 [22] was excluded as the dose response curve was abnormal (the drug became less effective at increasing concentrations) and not consistent with other antiviral studies with emetine.

Study	EC_{50} (μM) Emetine	Max. % Inhibition (Emetine conc., μM)*	SARS-CoV2 Strain
Choy *et al* 2020 [23]	0.46	~100% (<2)	BetaCoV/Hong Kong/VM20001061/2020, isolated from nasopharynx aspirate & throat swab of the first confirmed COVID-19 patient in Hong Kong
Bojkova *et al* 2020 [24]	0.47	~100% (2-5)	Isolated from samples of travellers returning from Wuhan (China) to Frankfurt (Germany)
Ellinger *et al* 2020 [25]	0.52	~70-75%** (~2)	Isolated from samples of travellers returning from Wuhan (China) to Frankfurt (Germany)

* Max % Inhibition (Emetine conc., μM) refers to the maximum % inhibition with emetine and the approximate lowest concentration this was achieved with, values were extracted from log graphs. **there are overlapping authors in Bojkova *et al* 2020 and Ellinger *et al* 2020. It is unknown if both studies used the same isolate of SARS-CoV2.

References

1. *The World Health Report 1998: life in the 21st century*; World Health Organization, Ed.; WHO: Geneva, 1998; ISBN 978-92-4-156189-1.
2. Juniper, K. Amebiasis in the United States. *Bull. N. Y. Acad. Med.* **1971**, *47*, 448–461.
3. Stanley, S.L. Amoebiasis. *Lancet Lond. Engl.* **2003**, *361*, 1025–1034, doi:10.1016/S0140-6736(03)12830-9.
4. Acton, H.W. Principles in the Testing of a Cure *Being a paper read at a meeting of the Medical Section of the Asiatic Society of Bengal on Monday, the 12th April, 1926. *Indian Med. Gaz.* **1926**, *61*, 271–279.
5. Harinasuta, C. A comparison of chloroquine and emetine in the treatment of amoebic liver abscess. *Indian Med. Gaz.* **1951**, *86*, 137–142.
6. *Pharmacological Basis Therapeutics 6th Ed ISBN: 978-0-02-344720-4*; Gilman Gilman, A., Goodman, L., Goodman, A., Eds.; Macmillan: New York, 1980;
7. Scragg, J.N.; Powell, S.J. Emetine hydrochloride and chloroquine in the treatment of children with amoebic liver abscess. *Arch. Dis. Child.* **1966**, *41*, 549–550, doi:10.1136/adc.41.219.549.
8. Klatskin, G. Observations on amebiasis in American troops stationed in India. *Ann. Intern. Med.* **1946**, *25*, 773–788, doi:10.7326/0003-4819-25-5-773.
9. Harriman, W.; Blaisdel, T. *Foreign commerce weekly Vol. 31 Issue 7 (May 15)*; Washington : U.S. Dept. of Commerce, 1948; p. 28;.
10. Stephen, P.M. Chapter 9: Cardiotoxicity of Emetine and Analogs. In *Principles of Cardiac Toxicology*; CRC Press, 1991 ISBN 978-1-4398-0536-7.
11. Grossman, W. Amebiasis in Peace Corps volunteers. Clinical profile of amebiasis in American volunteers in India. *Am. J. Gastroenterol.* **1969**, *51*, 418–433.
12. Census of India: Census Reports 1921 Available online: https://www.censusindia.gov.in/Census_And_You/old_report/Census_1921.aspx (accessed on Aug 10, 2020).
13. *Martindale: The Extra Pharmacopoeia 29th Edition 1989 ISBN: 978-0-85369-210-2*; Reynolds, J.E.F., Parfitt, K., Parsons, A., Sweerman, S., Eds.; 29th Edition.; The Pharmaceutical Press: London England, 1989; ISBN 978-0-85369-210-2.
14. Pedrazzoli, A.; Gradnik, B.; Dall’Asta, L. Amebicides. l-Emetine derivatives. *J. Med. Chem.* **1971**, *14*, 255–256, doi:10.1021/jm00285a025.
15. Burchard, G.D.; Mirelman, D. Entamoeba histolytica: Virulence potential and sensitivity to metronidazole and emetine of four isolates possessing nonpathogenic zymodemes. *Exp. Parasitol.* **1988**, *66*, 231–242, doi:10.1016/0014-4894(88)90095-1.
16. Bleasel, M.D.; Peterson, G.M. Emetine, Ipecac, Ipecac Alkaloids and Analogues as Potential Antiviral Agents for Coronaviruses. *Pharmaceuticals* **2020**, *13*, 51, doi:10.3390/ph13030051.
17. Asano, T.; Ishihara, K.; Wakui, Y.; Yanagisawa, T.; Kimura, M.; Kamei, H.; Yoshida, T.; Kuroiwa, Y.; Fujii, Y.; Yamashita, M.; et al. Absorption, distribution and excretion of 3H-labeled cephaeline- and emetine-spiked ipecac syrup in rats. *Eur. J. Drug Metab. Pharmacokinet.* **2002**, *27*, 17–27, doi:10.1007/BF03190401.
18. Parmer, L.G.; Cottrill, C.W. Distribution of emetine in tissues. *J. Lab. Clin. Med.* **1949**, *34*, 818–821.
19. Davis, B.; Dodds, M.G.; Tomich, E.G. Spectrophotofluorometric Determination of Emetine in Animal Tissues. *J. Pharm. Pharmacol.* **1962**, *14*, 249–252, doi:10.1111/j.2042-7158.1962.tb11087.x.
20. Schwartz, D.E.; Herrero, J. Comparative Pharmacokinetic Studies of Dehydroemetine and Emetine in Guinea Pigs Using Spectrofluorometric and Radiometric Methods*. *Am. J. Trop. Med. Hyg.* **1965**, *14*, 78–83, doi:10.4269/ajtmh.1965.14.78.

21. Tang, Q.; Li, S.; Du, L.; Chen, S.; Gao, J.; Cai, Y.; Xu, Z.; Zhao, Z.; Lan, K.; Wu, S. Emetine protects mice from enterovirus infection by inhibiting viral translation. *Antiviral Res.* **2020**, *173*, 104650, doi:10.1016/j.antiviral.2019.104650.

22. Weston, S.; Haupt, R.; Logue, J.; Matthews, K.; Frieman, M.B. FDA approved drugs with broad anti-coronaviral activity inhibit SARS-CoV-2 in vitro. *bioRxiv* **2020**, 2020.03.25.008482, doi:10.1101/2020.03.25.008482.

23. Choy, K.-T.; Yin-Lam Wong, A.; Kaewpreedee, P.; Sia, S.-F.; Chen, D.; Yan Hui, K.P.; Wing Chu, D.K.; Wai Chan, M.C.; Pak-Hang Cheung, P.; Huang, X.; et al. Remdesivir, lopinavir, emetine, and homoharringtonine inhibit SARS-CoV-2 replication in vitro. *Antiviral Res.* **2020**, 104786, doi:10.1016/j.antiviral.2020.104786.

24. Bojkova, D.; Klann, K.; Koch, B.; Widera, M.; Krause, D.; Ciesek, S.; Cinatl, J.; Münch, C. Proteomics of SARS-CoV-2-infected host cells reveals therapy targets. *Nature* **2020**, 1–8, doi:10.1038/s41586-020-2332-7.

25. Ellinger, B.; Bojkova, D.; Zaliani, A.; Cinatl, J.; Westhaus, S.; Reinshagen, J.; Kuzikov, M.; Wolf, M. Identification of inhibitors of SARS-CoV-2 in-vitro cellular toxicity in human (Caco-2) cells using a large scale drug repurposing collection [Under Review + supplementary files] DOI: 10.21203/rs.3.rs-23951/v1 https://www.researchsquare.com/article/rs-23951/v1. **2020**, doi:10.21203/rs.3.rs-23951/v1.
